# Carbohydrate Storage and Supplementation Strategies for Peak Performance in Cross‐Country Skiing

**DOI:** 10.1111/sms.70242

**Published:** 2026-02-26

**Authors:** Niels Ørtenblad, H.‐C. Holmberg, Lars Nybo, Kasper D. Gejl

**Affiliations:** ^1^ Department of Sports Science and Clinical Biomechanics University of Southern Denmark Odense Denmark; ^2^ Department of Health Sciences Luleå University of Technology Luleå Sweden; ^3^ School of Kinesiology University of British Columbia Vancouver British Columbia Canada; ^4^ Department of Physiology and Pharmacology Biomedicum C5, Karolinska Institutet Stockholm Sweden; ^5^ Department of Nutrition, Exercise and Sports University of Copenhagen Copenhagen Denmark

**Keywords:** biathlon, carbohydrate, cross‐country skiing, glycogen, Nordic combined, Olympic, performance, recovery

## Abstract

Carbohydrates are essential for sustaining performance in most competitive exercise, fueling both anaerobic glycolysis during high‐intensity efforts and aerobic metabolism during prolonged activity. Numerous factors contribute to muscle fatigue and exercise performance; still, carbohydrate and muscle glycogen contents are agreed to have an essential role in sustaining prolonged exercise at moderate‐to‐high intensities. To maintain consistent training and competition performance, elite athletes under certain conditions consume adequate carbohydrates between sessions to restore liver and muscle glycogen and possibly supplement during prolonged workouts to delay depletion. Effective glycogen restoration requires both sufficient carbohydrate intake and adequate recovery time. Understanding how glycogen levels fluctuate during intense or prolonged exercise, the rate at which stores are utilized, and the optimal amount and timing of carbohydrate intake for replenishment is essential. Here we examine the role of carbohydrate availability and utilization in competitive cross‐country skiing, which is characterized by exceptionally high whole‐body energy turnover with varying loads on the upper‐ and lower‐body muscles as well as fluctuating physiological demands determined by course profile, snow conditions, sub‐technique, and race format. This narrative review synthesizes existing evidence on the role of muscle glycogen contents and carbohydrate intake in muscle function and fatigue mechanisms, with a particular focus on cross‐country skiing and herein biathlon and Nordic combined. Additionally, we explore how exercise influences glycogen metabolism, the factors regulating glycogen utilization, and training adaptation in order to clarify physiological underpinnings and practical implications for endurance athletes.

## 1 Carbohydrate Availability and Performance

Among the many factors that influence the ability to sustain a given power output during exercise, the association between low muscle glycogen levels, carbohydrate availability, and fatigue, has probably been most universally acknowledged (for a more comprehensive review see [1]). Thus, carbohydrates are fundamental for supporting high‐intensity exercise performance, providing the substrate for rapid ATP resynthesis during short maximal efforts dependent on anaerobic glycolysis, as well as during prolonged intense exercise characterized by high rates of carbohydrate oxidation [2]. Beyond sustaining both brain and muscle metabolism, intramuscular carbohydrate stores (i.e., muscle glycogen located within and between myofibrils) play a crucial role in maintaining the individual muscle fiber capacity to generate force by influencing key processes involved in the muscle contraction [3]. Thus, depletion of the restricted muscle glycogen stores markedly reduces the metabolic capacity within muscle cells, thereby impairing the energy supply needed to sustain muscle contraction and power production, eventually causing decreased exercise performance [1].

**FIGURE 1:**
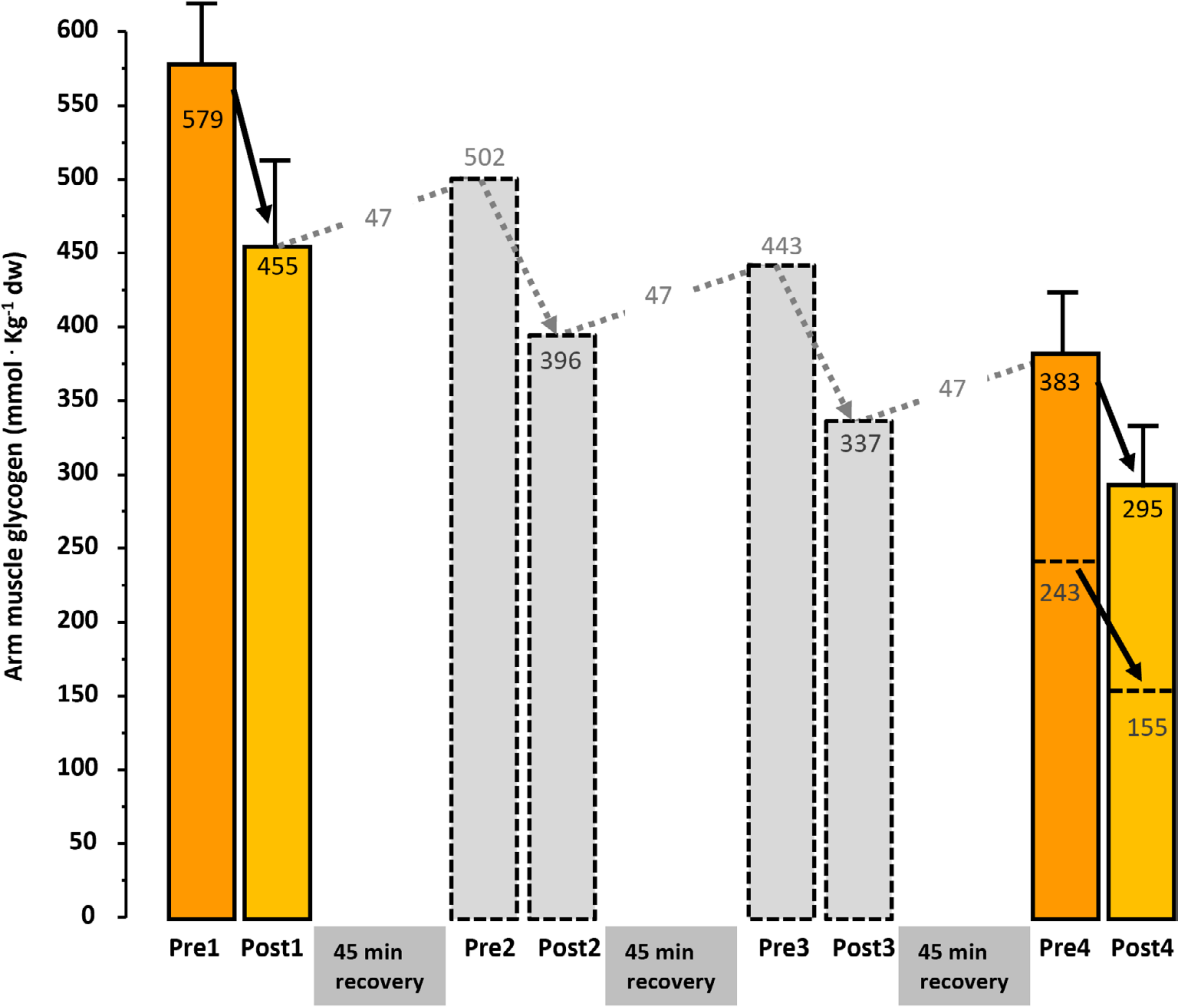
Muscle glycogen utilization and estimated resynthesis in arm muscle (*m. triceps brachii*) during four repeated 1300‐m highintensity skiing bouts. Elite cross‐country skiers fulfilled four successive 1300‐m high‐intensity skiing bouts (~4 min), interspersed with 45‐min recovery periods during which 70 g carbohydrate were consumed. Glycogen decreased from 579 to 455 mmol·kg^−1^ dw during sprint 1 (21%) and from 383 to 295 mmol·kg^−1^ dw during sprint 4 (23%) (dark bars pre‐post, 1 and 4). Assuming similar 21%–22% utilization during sprints 2 and 3, the glycogen resynthesis rate can be predicted to be 140 mmol during the three 45‐min recovery periods (i.e., ~47 mmol per period or ~63 mmol h^−1^), see text (pre‐post, 2 and 3). Furthermore, without resynthesis, the projected post‐sprint‐4 glycogen concentration would have been 155 mmol (indicated by dotted lines), that is, levels significantly below the apparent threshold for glycogen being limiting for muscle function and performance. Parts of the data shown in this figure are derived from Gejl et al. [10] and partly from unpublished observations.

It is important to recognize that numerous factors contribute to muscle fatigue and exercise performance; still, carbohydrate and muscle glycogen contents are in most conditions agreed to have an essential role in sustaining prolonged exercise at moderate‐to‐high intensities [1, 4]. Carbohydrate oxidation provides energy both more rapidly and more efficiently than fat oxidation. Thus, under aerobic conditions, carbohydrate combustion is a more efficient fuel yielding approximately 21.1 kJ/L O_2_, whereas fat oxidation yields ~19.6 kJ/L O_2_ [5]. In addition, anaerobic glycogen breakdown to lactate and aerobic carbohydrate metabolism allows muscles to generate ATP at rates 3**–**4 times faster than aerobic carbohydrate combustion and roughly seven times faster than fatty acid metabolism [6, 7]. These characteristics highlight the critical role of carbohydrates in fueling repeated high‐intensity efforts, explaining the predominance of carbohydrate oxidation at the intensities (> 75% V̇O_2_max) sustained in most competitive events, including cross‐country skiing and biathlon [8, 9]. This also clarifies why glycogen utilization is extremely high during repeated maximal bouts of exercise (Ref. [1]; see also Figure 1). Muscle glycogen use increases disproportionately with rising exercise intensity at workloads near or above V̇O_2_max [11]. For instance, a single 75‐s all‐out effort can reduce muscle glycogen by ~20% [12], whereas a 15‐min maximal effort has been shown to lower glycogen availability by ~30% [13]. Given that many cross‐country skiing formats involve repeated high‐intensity surges interspersed with lower‐intensity periods, glycogen depletion may occur more rapidly and more extensively than during continuous exercise protocols typically used in laboratory settings.

Beyond serving as a metabolic substrate during intense exercise, muscle glycogen is also essential for maintaining contractile function by regulating key processes involved in skeletal muscle activation. Thus, low muscle glycogen levels influence both force and power production by modulating muscle activation through effects on muscle excitability and sarcoplasmic reticulum Ca^2+^ release rate [3, 14, 15], which becomes evident when muscle glycogen concentration falls below an apparent level of 250–200 mmol kg^−1^ dw in most [15, 16, 17], but not all studies [18]. Consequently, when individual fibers become glycogen‐depleted, fatigue manifests as a reduced ability to generate maximal force or power, driven by impaired function within the affected myofibrils.

## 2 Physical Demands in Cross‐Country Skiing

Competitive cross‐country ski races are characterized by high whole‐body energy turnover, varying loads on upper‐ and lower‐body muscles, and fluctuating physiological demands determined by course profile, snow conditions, sub‐technique, and race format [19]. Events range from repeated high‐intensity sprints to endurance races lasting several hours. Individual sprint competitions involve a qualification time trial followed by three knockout heats (quarter‐final, semi‐final, and final) of ~2.5–3.5 min duration, where the recovery time gradually reduces to ~15–30 min as the competition progresses. The team sprint is a relay event where two skiers alternate multiple short laps of the same time as the individual sprint, with each skier completing three sprints separated by brief recovery periods while their teammate races. Longer Olympic‐distance events range from ~20–50 min for the 10 individual and 20 km Skiathlon (10 + 10 km) races to ~2–2½ h for the 50‐km mass start [20, 21]. Noteworthy, with the recent equalization of race distances, men and women compete on identical courses, resulting in ~10%–15% longer race durations for female skiers—a factor directly influencing substrate utilization.

In Olympic and World Cup races, athletes spend ~35% of race time on flat sections, ~15% on descents, and ~50% on uphill sections [22]. Notably, the latter poses the highest physiological load and constitutes the performance‐discriminating segments [21, 22]. This terrain‐driven “micro‐pacing” implies elevated work rates on climbs and opportunity for partial recovery on descents, thereby yielding an energetic signature that couples high sustained aerobic turnover with repeated supramaximal bursts (eliciting ~120%–160% of V̇O_2_peak on steep uphill sections) [22, 23]. The intermittent work pattern will be scenario‐specific for the given race and will depend on the course profile, but with the mean aerobic contribution estimated at **~**70%–75% in sprints and 85%–95% in distance events. In addition, the instantaneous contribution from anaerobic energy systems during climbs (followed by recovery on descents and drafting in mass starts) or during the end‐spurt may account for up to ~40% in sprint events and 10%–20% in longer distance races [9, 22].

Given these intermittent demands, the concepts of durability and physiological resilience [24] are critical for understanding skiing performance. These terms describe the ability to maintain exercise economy and fractional energy utilization despite accumulating fatigue and include the various subcomponents of performance (i.e., exercise economy, peak aerobic and anaerobic energy contribution and fractional utilization of these energy systems) are affected over the time‐course of the competition. In the context of this review, this includes how carbohydrate ingestion may impact the ability to prevent the degradation of these parameters [25] and impact the ability to repeat intense efforts throughout the race, recovery from day to day or from heat to heat in the sprinting events. For cross‐country skiing it is characteristic that movement efficiency depends on technical capabilities as well as biomechanical and metabolic properties, with carbohydrate availability obviously of relevance for the metabolic as discussed below. Also, depletion of muscle glycogen in specific fibers may impact peak anaerobic power secondary to lowered glycolytic flux. Hence, specific components of durability may be affected over the time‐course of a race and potentially affected by the pre or in race carbohydrate fueling strategy. Fatigue secondary to loss of muscle fiber function may further impact the skiers technical efficiency, stride length, cycle rate, and force–time characteristics, while loss of anaerobic power is relevant for race performance, particularly on climbs where mechanical power must be increased for optimal pacing [19, 23, 26, 27]. Skiers here use sub‐technique selection as a “gearing system” governing upper‐ and lower‐body contribution and metabolic cost as speed and gradient change; elite skiers transition frequently (~25 transitions km^−1^) as an attempt to maintain economy and force application across variable terrain [19, 22, 23, 28]. However, this also imposes distinct metabolic loads on specific muscle group—particularly in double poling, which alters cardiorespiratory and neuromuscular demands, reinforcing the sport's status as a whole‐body endurance model that taxes both central O_2_ delivery and challenge peripheral metabolic energy turnover [19, 29].

Physiologically, elite skiers exhibit some of the highest documented aerobic capacities: V̇O_2_max ≈80–90 and 70–80 mL kg^−1^ min^−1^ for men and women, respectively; maximal ventilation up to ~200–250 L min^−1^; stroke volumes exceeding 200 mL; and cardiac output greater than 35–40 L min^−1^ during race‐specific exercise [19, 20]. Race intensity typically averages ~84%–90% of V̇O_2_peak across varied terrain, with elevated blood lactate reflecting the intermittent supramaximal surges superimposed upon a predominantly aerobic event [22, 23]. Elite cross‐country skiers, who are characterized by equally trained upper‐ and lower‐body, arm and leg muscles, have been shown to exhibit similar mitochondrial volume fractions, capillarization, and citrate synthase activity, despite a higher proportion of MHCII fibers in the arms [30]. This indicates a dissociation between fiber‐type distribution and aerobic metabolic capacity. In contrast, marked limb‐specific differences in fatty‐acid–oxidative capacity (HAD activity), together with a fourfold higher intramyocellular lipid content in leg muscles, reveal distinct metabolic characteristics not explained by fiber type composition [31].

In summary, the physiological demands of elite cross‐country skiing (and similarly of the endurance component of biathlon and Nordic combined) include: (i) exceptionally high V̇O_2_peak and efficiency across multiple sub‐techniques; (ii) the capacity for repeated supramaximal bursts; and (iii) the physiological resilience to maintain technical precision and tactical execution under metabolic fatigue [8, 19, 20, 22].

## 3 Carbohydrate Utilization During Cross‐Country Skiing

The glycogen utilization during cross‐country skiing depends on course profile, snow conditions, sub‐technique, and race format, all of which influence the relative metabolic load placed on upper‐ versus lower‐body musculature. Of note, glycogen is typically reported as a concentration (mmol kg^−1^ dw). However, because the active upper‐body muscle mass is substantially smaller than the lower‐body muscle mass, the arms have a lower absolute glycogen storage capacity even when concentrations are comparable, which may increase susceptibility to local glycogen depletion when upper‐body work is emphasized.

Data regarding glycogen utilization in cross‐country skiers remain scarce. Two studies have examined muscle glycogen consumption in recreational skiers during the “Vasaloppet” (85 km at that time) finished at on average ~7 h 30 min [32, 33], a race characterized by a substantial contribution of double poling. Arm glycogen stores were depleted 86% [32], compared to an average reduction of 58% in the legs [32, 33]. At the World Cup level, a 90% reduction in muscle glycogen levels was observed following a 50 km ski race, and glycogen stores decreased to 50% following 10 (female) and 15 km (male) ski races [34]. In a shorter, ~1 h ski race with junior elite cross‐country skiers, involving mainly double poling technique, glycogen was on average reduced to 31% (from 540 to 167 mmol kg^−1^ dw) in the arms and 71% (from 485 to 331 mmol kg^−1^ dw) in the legs directly after the race [15]. During more intense formats of sprint skiing, glycogen utilization is substantially higher. This has been shown in a study on elite cross‐country skiers completing four successive 1300‐m high‐intensity skiing bouts (~4 min), interspersed with 45‐min recovery periods during which 70 g carbohydrate were consumed [10, 35]. Muscle glycogen was measured in arm (*m. triceps brachii*) and legs (*m. vastus lateralis*) before and after bout 1 and bout 4 to evaluate glycogen utilization and resynthesis across the session (Figure 1). There was a similar relative glycogen utilization in bout 1 and 4, with an average glycogen utilization of 22% in arms and 18% in legs, reflecting the relative contribution of upper and lower body. Assuming bouts 2 and 3 produced similar relative glycogen costs in arm, an average utilization of 106 mmol per bout can be estimated, resulting in a calculated glycogen concentration of 155 mmol kg^−1^ dw following bout 4 with no carbohydrate consumption during recovery, i.e., levels significantly below the apparent threshold at which glycogen limits muscle function (see *Carbohydrate availability and performance*). With the carbohydrate intake of 70 g carbohydrate during the three 45‐min recovery periods between sprints, there was an estimated average glycogen resynthesis rate of ~47 mmol per period or ~63 mmol h^−1^. Together, this underscores the importance of carbohydrate supported recovery to maintaining glycogen availability, even across four repeated short high‐intensity heats as in sprint skiing. In leg muscle, glycogen contents were 303 mmol kg^−1^ dw after bout 4, which with same calculations approach would be 269 mmol kg^−1^ dw without carbohydrate intake. Importantly, sprint skiing also includes considerable exercise during ski testing before the race, warm‐up, and cool‐down periods. Indeed, a recent analysis of a sprint competition revealed that skiers cover a total distance of ~25 km and perform ~2 h of low‐to‐moderate intensity exercise in addition to the high‐intensity heats [36]. This underscores the critical role of carbohydrate‐fueling strategies even within the sprint‐skiing format.

Collectively, these studies illustrate that glycogen utilization is highly dependent on exercise intensity and duration during cross‐country skiing. Glycogen utilization rates varied from 21% (arm) and 16% (leg) during a 4 min sprint; 66% (arm) and 39% (leg) during a 1‐h ski race; to 86% (leg) during a race lasting 7 h 30 min in recreational skiers. This corresponds to approximately (mmol glycogen kg^‐1^ min^‐1^) 25 during a 4‐min sprint (arm and leg), 4.4 during a 1‐h race (arm and leg), and 0.8 during a prolonged race as during the Worldloppet series, demonstrating the pronounced role of exercise intensity in glycogen utilization.

Sex differences in substrate metabolism are evident, although direct comparisons present methodological challenges. Biologically, females typically exhibit a lower reliance on whole‐body carbohydrate metabolism to support energy production during endurance exercise and although findings are not entirely consistent, several studies also indicate that females utilize less glycogen than males. In cross‐country skiing, these sex differences are further modulated by different exercise loads on specific muscle groups. At matched relative intensities, women utilize diagonal stride more frequently than men, who rely more on double poling [21, 28]. Furthermore, due to the equalization of race distances, female skiers typically experience ~10%–15% longer race durations than males. These differences in technique distribution and absolute duration possibly alter the specific muscle loading patterns and total energy expenditure required for competition. However, the absolute difference in glycogen utilization between sexes is considered small [37] and from a practical standpoint, we therefore suggest that the magnitude of these sex differences is unlikely to justify sex‐specific nutritional strategies [1]. Rather, both males and females should ensure that they begin training sessions or competitive events with sufficient glycogen stores to meet the expected metabolic demands.

Alongside the role of glycogen in muscle function and exercise performance, strategies such as glycogen supercompensation [38] and carbohydrate ingestion during exercise have consistently been shown to improve performance during intense efforts lasting more than 1 h and prolonged exercise exceeding 2.5 h [39]. The ergogenic effect of carbohydrate feeding under such conditions is primarily mediated by the maintenance of plasma glucose, the preservation of whole‐body carbohydrate oxidation rates, and the sparing of liver glycogen, all of which play a critical role in sustaining performance and endurance capacity [38, 40]. The following sections address carbohydrate intake before, during, and in the recovery from exercise, focusing on strategies to delay glycogen depletion, reduce fatigue, and enhance performance. It also briefly considers how environmental factors and carbohydrate availability interact with training adaptations and substrate utilization.

## 4 Pre‐Exercise Carbohydrate Availability: Striking the Right Balance

As denoted, competitive cross‐country skiing events impose substantial demands on both aerobic and anaerobic energy systems, involving high carbohydrate turnover and an increased risk of limited carbohydrate availability that may ultimately constrain performance. Although endogenous carbohydrate stores—primarily liver glycogen and muscle glycogen—can become critically depleted, optimal fueling is not simply about maximizing glycogen stores. Pre‐competition nutrition should balance sufficient energy provision with the need to avoid unnecessary increases in body mass with excessive glycogen and its accompanying water storage [13]. Therefore, a carefully tailored carbohydrate intake strategy before, during, and after competition is essential to support optimal performance.

Muscle glycogen is a finite but critical substrate for sustaining the frequent high‐intensity efforts characteristic of cross‐country skiing. For events in which carbohydrate availability may become critical, e.g., Vasaloppet, Worldloppet series, World Cup and Olympic 50 km race or the individual or team sprint format with repeated bouts [10, 32], strategies to increase pre‐competition glycogen stores can therefore confer performance benefits [38]. This is also important to consider for multi‐day skiing competitions, such as those held during the Olympics, World Cup, and Tour de Ski, where carbohydrate intake and recovery duration can be limited. Endogenous carbohydrate reserves are highly adaptable, with endurance training enhancing the muscle glycogen storage capacity, resulting in trained athletes having approximately twice as high muscle glycogen concentration as regular trained [37]. Beyond this training adaptation, muscle glycogen availability can be augmented above normal levels (i.e., “super‐compensated”) through dietary strategies involving high carbohydrate intake [41]. While early carbohydrate‐loading protocols incorporated a glycogen depletion phase followed by high carbohydrate consumption [42], contemporary approaches demonstrate that similar levels of glycogen supercompensation can be achieved without the necessity of prior depletion [41, 43]. Current guidelines for maximal glycogen loading recommend an intake of 8–12 g kg^−1^ body mass per day for 36–48 h preceding competition [38, 41, 44], often combined with a lower exercise load. While this recommendation is primarily relevant for the 50 km race format or prolonged events like Vasaloppet, during which glycogen depletion may directly limit performance, less aggressive and more event‐specific loading strategies may be more suitable for shorter events.

Although insufficient glycogen leads to premature fatigue and reduced power output, excessive loading can also be counterproductive due to an associated increase in body mass. Glycogen is stored in muscle with water in an approximate 1:3 ratio, meaning that glycogen loading has direct consequences for body mass [13, 45]. In highly trained endurance athletes, muscle glycogen concentrations can reach 700–800 mmol kg^−1^ dw, which corresponds to water retention equivalent to as much as ~3 to 4 L for a 75 kg athlete [13, 46]. Thus, excessive glycogen storage is accompanied by substantial increases in non‐functional mass, which may compromise performance in weight‐bearing activities where movement efficiency is key [13, 47]. Consequently, athletes with a high capacity for glycogen storage may benefit from moderately reducing pre‐event carbohydrate intake relative to maximal loading before shorter cross‐country skiing events (e.g., sprint, 10 km, skiathlon, and relays). This approach may lower body mass by 1–2 kg, thereby improving movement economy and power‐to‐weight ratio [13, 48]. Nevertheless, such individualized strategies should be carefully tested in training, as overly conservative fueling increases the risk of early fatigue and suboptimal race performance.

## 5 Pre‐Race Meals: Timing and Composition

In addition to total endogenous glycogen availability, ensured via the above guidelines, consumption of a carbohydrate‐enriched pre‐race meal can further enhance performance [49]. In addition to increasing muscle glycogen content further [50, 51, 52], a carbohydrate‐enriched pre‐race meal also supports the maintenance of blood glucose and liver glycogen concentrations [44, 53], which become depleted by fasting (e.g., overnight prior to race) [54]. Depending on the metabolic demands of the event, the current guidelines recommend ingesting 1–4 g carbohydrate kg^−1^ body mass approximately 3–4 h before competition, with an optional “top‐up” of ~1 g kg^−1^ body mass within the final hour before competition [38]. This is particularly relevant for multi‐day skiing competitions, where carbohydrate intake can be limited by short recovery time between competitions.

The glycemic index (GI) of the pre‐exercise meal has been proposed to influence substrate utilization and performance [55]. Specifically, low‐GI meals promote more stable blood glucose and insulin levels, facilitating greater reliance on fatty acid oxidation, and potentially sparing muscle glycogen stores [56]. Conversely, a high‐GI meal may cause a rapid rise in blood glucose and insulin, sometimes followed by transient hypoglycemia near the onset of exercise [52]. Nonetheless, evidence suggests that GI of the pre‐event meal has little impact on performance in events where continuous exogenous carbohydrate intake is feasible [55]. In contrast, a low‐GI pre‐exercise meal may offer a performance advantage for prolonged competitions with limited opportunities for carbohydrate ingestion (e.g., 50 km mass start). Importantly, the timing and composition of the pre‐competition meal should also account for gastrointestinal comfort and individual tolerance. In this context, substantial inter‐individual variability exists in glycemic responses to the same carbohydrate‐rich foods, indicating that GI is influenced not only by food properties but also by personal metabolic and physiological factors [57].

## 6 Carbohydrate Intake During Cross‐Country Skiing

Nutritional recommendations for endurance athletes have primarily been derived from studies utilizing cycling or running protocols performed under steady‐state exercise conditions, typically in moderately and highly trained athletes [58]. In contrast, cross‐country skiing events present unique physiological demands, involving simultaneous activation of both upper‐ and lower‐body musculature, a high energy turnover, and highly variable intensity with substantial intermittent anaerobic contributions, factors which significantly increase carbohydrate requirements [23]. Furthermore, as cross‐country skiing is predominantly performed in cold environments, carbohydrate oxidation rates may be further elevated compared with exercise in temperate conditions [59]. Despite these specific demands, direct evidence regarding nutritional strategies derived from elite cross‐country skiers remains limited [60, 61], which highlights a clear need for more specific investigations to refine nutritional recommendations in this population.

Carbohydrate requirements and glycogen utilization during racing depend on duration and its interaction with intensity [39]. For short cross‐country skiing events lasting up to 45 min, endogenous glycogen stores are generally sufficient, and additional carbohydrate intake is unlikely to provide performance benefits unless the event commences with suboptimal glycogen availability due to repeated exercise bouts with inadequate time for glycogen resynthesis and/or multi‐day skiing competitions [38]. As stated above, carbohydrate metabolism accounts for nearly 100% of energy provision during high‐intensity races, including intermittent race mode, which is mainly covered by high rates of muscle glycogen degradation, demonstrated in both males and females [62, 63]. Thus, a higher energy demand due to higher exercise intensity does not increase energy contribution from blood glucose or fatty acid, but is met from a greater utilization of muscle glycogen, with muscle glycogen contributing about 70% of the energy expenditure when exercising at 85% V̇O_2_peak [64].

In moderate‐duration cross‐country skiing events (45–75 min), ingestion of small amounts of carbohydrate can help maintain blood glucose levels and attenuate central fatigue [39]. Consequently, carbohydrate recommendations for such events are in the lower end of the intake range (i.e., ~20–30 g h^−1^), with individualized training and testing used to determine tolerable and effective amounts.

For events longer than 75 min, carbohydrate intake becomes increasingly important, with current guidelines recommending progressively higher intakes with increased duration (scaling from 30 to 90 g h^−1^) to prevent liver glycogen depletion and to maintain blood glucose concentrations and carbohydrate oxidation, despite progressive reductions in muscle glycogen availability [38]. Consequently, intakes of up to 90 g h^−1^ in a 1:0.8 glucose‐fructose ratio are advised for competitions such as the 50 km race format (~120–150 min) [38, 58, 65].

Glucose absorption occurs via the sodium‐glucose cotransporter 1 (SGLT1), which has been suggested to reach saturation at ~60 g h^−1^ with peak exogenous carbohydrate oxidation rates of ~1.0–1.1 g carbohydrate min^−1^ [40], whereas fructose is absorbed independently through the glucose transporter 5 (GLUT5) [66], resulting in peak exogenous carbohydrate oxidation rates of ~1.3 g carbohydrate min^−1^ [67]. Besides greater total carbohydrate absorption and oxidation rates, glucose‐fructose mixtures also reduce the risk of gastrointestinal discomfort [68].

As outlined previously, the specific demands of cross‐country skiing (i.e., complex muscle activation patterns, several sub‐techniques, and variable exercise intensities) may increase reliance on exogenous carbohydrate compared with other endurance activities. Furthermore, carbohydrate intake recommendations are derived from studies in moderately to highly trained individuals rather than elite athletes, who exhibit higher absolute energy turnover and an elevated glucose flux to the working muscles. Consequently, although current guidelines advise up to 90 g h^−1^ during prolonged events, it is unclear if even higher intake rates may confer additional benefits for elite cross‐country skiers. Anecdotal reports from elite endurance athletes indicate that carbohydrate intakes of 100–180 g h^−1^ is consumed to maximize exogenous carbohydrate availability and sustain energy delivery during competition. Supporting this notion, a recent study in trained males demonstrated greater exogenous carbohydrate oxidation rates during prolonged steady‐state exercise when ingesting 120 g h^−1^ compared with 90 g h^−1^ (1:0.8 glucose‐to‐fructose ratio), corresponding to oxidation rates of 1.5 vs. 1.3 g min^−1^, respectively [67]. While these findings indicate that exogenous carbohydrate oxidation can be augmented by doses beyond the current recommendations, the performance implications for elite cross‐country skiers performing intermittent activities within the heavy and severe exercise domains remain uncertain. Moreover, while recommended carbohydrate doses during exercise has been shown spare liver‐ and muscle glycogen [69, 70, 71], it remains uncertain how very high doses of carbohydrate (> 100 g/h) affects each of the endogenous carbohydrate pools during prolonged intermittent exercise in elite athletes.

Together, for prolonged events and based on existing evidence, carbohydrate intakes approaching 90 g h^−1^ may help sustain high carbohydrate oxidation rates and support performance in the later stages of prolonged cross‐country skiing competitions. However, in practice frequent intake may interfere with poling, strategic positioning, achievability, aerodynamics, and safety, necessitating strategic timing and methods to ingest carbohydrate, based on the course profile and race dynamics. A practical approach during prolonged events is to consume carbohydrate approximately every 20 min (i.e., ~25–30 g per intake), in addition to carbohydrate mouth rinsing during the latter stages of the race, when carbohydrate availability may become critical (see *Carbohydrate Mouth Rinsing During Competition*) [72].

Importantly, high doses of exogenous carbohydrate are associated with an increased risk of gastrointestinal (GI) symptoms such as nausea, bloating, and diarrhea [73]. To minimize these risks, athletes are advised to progressively habituate to high carbohydrate intake during training, a process referred to as “gut training” [74]. Regular exposure to high carbohydrate loads may upregulate SGLT1 expression, enhance intestinal absorptive capacity, reduce GI distress, and ultimately increase the capacity for exogenous carbohydrate oxidation [74, 75, 76].

## 7 Carbohydrate Mouth Rinsing During Competition

During periods of exercise where carbohydrate availability is critically reduced, carbohydrate mouth rinsing (e.g., 5–10 s every 5th min with a 6% carbohydrate solution) has been shown to enhance performance via a non‐metabolic mechanism [39, 72]. This ergogenic effect is thought to arise from the stimulation of carbohydrate‐sensitive receptors in the oral cavity, which activate brain regions involved in motor control and motivation [77, 78]. Although mouth rinsing could potentially improve performance during the later stages of a sprint competition or the 50 km mass start, when carbohydrate availability may become low [10], frequent mouth rinsing is on the other hand impractical, and maintaining a continuous presence of carbohydrate in the oral cavity through solid foods (e.g., wine gums) may represent a feasible alternative strategy.

## 8 Post‐Exercise Recovery

For the timely recovery of muscle function, rapid glycogen restoration is critical following competition, during recovery between repeated heats in sprint disciplines, or during periods of frequent high‐intensity training. Glycogen synthesis is most rapid in the immediate post‐exercise period, and a carbohydrate intake of 1.0–1.2 g kg^−1^ body mass per hour is recommended during the first 4–6 h after glycogen‐depleting exercise [58]. To maximize glycogen resynthesis and minimize gastrointestinal discomfort, distributing carbohydrate across small frequent snacks may be advantageous [58]. Combining carbohydrates with protein in a 3:1 ratio can further accelerate recovery, provided a suboptimal carbohydrate intake and support muscle repair [41].

## 9 Periodized Carbohydrate Availability in Training—Metabolic Stressors

While carbohydrate intake during competition and periods of high‐intensity training primarily aims to maximize performance, training also offers opportunities to manipulate substrate availability to promote specific physiological adaptations (e.g., “train‐low” or “metabolic‐stress”) [79, 80]. Train‐low strategies involve deliberately reducing carbohydrate availability around selected training sessions to enhance molecular signaling pathways associated with mitochondrial biogenesis [64]. Training under conditions of low muscle glycogen availability has been shown to acutely augment the activation of AMP‐activated protein kinase (AMPK) and p38 mitogen‐activated protein kinase (MAPK) [80]. These kinases can subsequently activate and promote the nuclear and mitochondrial translocation of peroxisome proliferator‐activated receptor gamma coactivator 1‐alpha (PGC‐1α), a central regulator of mitochondrial proliferation and oxidative capacity [81]. Consequently, repeated “train‐low” sessions have been hypothesized to enhance skeletal muscle oxidative capacity and improve endurance performance compared to training with ample carbohydrate availability. Despite promising acute molecular responses, training studies have provided limited and inconsistent evidence that carbohydrate periodization translates into measurable improvements in performance [82]. However, it is important to note that existing studies in trained individuals have been relatively short in duration (i.e., 3–4 weeks), leaving uncertainty as to whether longer‐term exposure to periodized low‐carbohydrate strategies could yield performance benefits. Importantly, exercising with low carbohydrate availability may elevate perceived exertion, reduce achievable training intensity, increase the risk of fatigue and impair immunological function, if applied excessively [83, 84, 85]. Therefore, periodized carbohydrate restriction should be implemented cautiously, targeted to specific sessions, and combined with sufficient recovery and adequate carbohydrate intake to support high‐intensity training on other days.

## 10 Perspective

Carbohydrate availability and muscle glycogen levels are consistently recognized as an essential fuel source to sustain high metabolic power output during exercise. Although many factors influence muscle fatigue and performance, carbohydrate availability should be considered across cross‐country skiing disciplines. Fueling strategies should reflect race duration and intensity of the event. If well rested, a moderate pre‐race carbohydrate intake is sufficient for most race formats. However, carbohydrate availability may become critical in the individual or team sprint format with repeated high‐intensity bouts as well as longer ski races as the Olympic 50 km race and especially for multi‐day skiing competitions, such as those held during the Olympics, World Cup, and Tour de Ski, where carbohydrate intake and recovery duration can be limited. Under these conditions, effective glycogen resynthesis requires adequate carbohydrate intake and sufficient recovery duration. It is also important to note that excessive carbohydrate intake before competition can lead to unnecessary increases in body mass due to the associated water storage with glycogen, impairing the power‐to‐weight ratio in shorter events. Overall, carbohydrate intake before and during competition should be carefully aligned with the demands of exercise to support sustained intensity and optimize skiing performance.

## 11 Summary and Practical Implications

- Carbohydrate availability is important across all cross‐country skiing disciplines. Fueling strategies should match race duration and intensity, with the understanding that endogenous stores (i.e., muscle and liver glycogen) are the primary sources of energy in all events.
- Excessive carbohydrate intake before events can lead to unnecessary increases in body mass due to the storage of ≈3 g water per gram of glycogen. This added mass may impair power‐to‐weight ratio, movement economy, and climbing ability in shorter events.
- If well rested, a moderate pre‐race carbohydrate intake is sufficient for all race formats except the 50‐km mass start, where maximal glycogen loading remains beneficial.
- For prolonged events, 60–90 g h^−1^ of carbohydrate helps sustain energy output; however, race strategies must also consider practical constraints and individual gastrointestinal tolerance.
- Post‐exercise recovery including ≈1–1.2 g kg^−1^ h^−1^ of carbohydrate, particularly in the hours immediately following training or competition, optimizes glycogen resynthesis ahead of subsequent sessions or races.
- All nutritional strategies should be thoroughly practiced in training, including gastrointestinal “gut training,” to enhance tolerance, absorption, and race‐day reliability.

## Funding

Team Denmark by means from the Novo Nordisk Foundation to the Danish Elite Endurance Performance Network.

## Conflicts of Interest

The authors declare no conflicts of interest.

## Data Availability

The data that support the findings of this study are available on request from the corresponding author. The data are not publicly available due to privacy or ethical restrictions.
